# Genotype-phenotype heterogeneity among patients with lipodystrophy harboring rare *POLD1* variants

**DOI:** 10.1210/clinem/dgag079

**Published:** 2026-02-25

**Authors:** Fieke W Hoff, Chao Xing, Chun-Yuan Huang, Vinaya Simha, Rebecca J Brown, Abhimanyu Garg

**Affiliations:** Hematology Branch, National Heart, Lung, and Blood Institute, National Institutes of Health, Bethesda, MD 20892, USA; Eugene McDermott Center for Human Growth and Development, UT Southwestern Medical Center, Dallas, TX 75390-8591, USA; Lyda Hill Department of Bioinformatics, UT Southwestern Medical Center, Dallas, TX 75390, USA; O’Donnell School of Public Health, UT Southwestern Medical Center, Dallas, TX 75390-9066, USA; Eugene McDermott Center for Human Growth and Development, UT Southwestern Medical Center, Dallas, TX 75390-8591, USA; Division of Endocrinology, Department of Internal Medicine, Mayo Clinic, Rochester, MN 55905, USA; National Institute of Diabetes and Digestive and Kidney Diseases, National Institutes of Health, Bethesda, MD 20892, USA; Section of Nutrition and Metabolic Diseases, Division of Endocrinology, Department of Internal Medicine and the Center for Human Nutrition, UT Southwestern Medical Center, Dallas, TX 75390-8537, USA

**Keywords:** mandibular hypoplasia, deafness, progeroid features, lipodystrophy, MDPL, POLD1

## Abstract

**Context:**

Mandibular hypoplasia, deafness, progeroid features, and lipodystrophy (MDPL) syndrome is a rare, autosomal dominant disorder due to pathogenic heterozygous variants in *POLD1*. Clinical features of MDPL vary between patients; however, there is no previously reported genotype-phenotype association.

**Objective:**

This work reports 14 new patients with lipodystrophy due to *POLD1* variants and compares phenotypic differences between those with p.Ser605del and missense variants.

**Methods:**

Genetic sequencing was performed on DNA of 14 patients for *POLD1* variants, including exome (n = 10), genome (n = 1), and candidate gene (n = 3) sequencing. Comparisons of demographic, clinical features, and metabolic complications between carriers of *POLD1* p.Ser605del and missense variants in our cases and those reported in the literature were made using the Fisher exact test for categorical variables and the *t* test for continuous variables.

**Results:**

A total of 9 different *POLD1* variants were identified in our patients, including 3 novel variants: p.Asp25Glufs*16, p.Arg507His, and p.Trp781Cys. Compared to individuals with missense variants (n = 15), those with the p.Ser605del (n = 26) *POLD1* variant had significantly increased prevalence of mandibular hypoplasia (57% vs 100%, respectively; *P* = .015), small mouth (36% vs 100%, respectively; *P* = .015), crowded teeth (44% vs 91%, respectively; *P* = .046), and hypogonadism in male patients (0% vs 92%, respectively; *P* = .046). There were no differences in the prevalence of metabolic complications, such as diabetes, hypertriglyceridemia, and hepatic steatosis, in the two groups.

**Conclusion:**

Individuals with the heterozygous *POLD1* p.Ser605del variant had typical MDPL with more severe phenotype compared to those with missense variants with atypical MDPL.

Mandibular hypoplasia, deafness, progeroid features, and lipodystrophy (MDPL) syndrome is a rare, autosomal dominant disorder characterized by prominent loss of subcutaneous fat, a facial appearance characteristic of premature aging, metabolic abnormalities, and sensorineural deafness that occurs in the first or second decade of life (1). It was first described in 2010 by our group in 7 patients with a remarkably homogeneous phenotype (1). Subsequently, Weedon et al (2) reported a recurrent de novo heterozygous c.1812_1814delCTC (p.Ser605del) variant in the DNA polymerase δ 1, catalytic subunit (*POLD1*) gene in 4 patients (including 2 patients previously reported by us) that affected the polymerase activity of POLD1. Since then, a total of 33 patients with pathogenic variants in *POLD1* have been published, with 25 harboring the same heterozygous c.1812_1814delCTC (p.Ser605del) variant (3-13), and 8 patients with other heterozygous missense variants (3, 7, 14-18). Most variants, but not all, were de novo. While some phenotypic variability has been reported among patients harboring the p.Ser605del variant, whether there is any robust genotype-phenotype association resulting in phenotypic variability remains unclear (15). For example, whether those with missense *POLD1* variants present with milder phenotype than those with the p.Ser605del variant remains unclear due to the small number of patients with missense variants reported so far. Therefore, we report genotype-phenotype relationships in a cohort of 14 patients, who primarily presented to us with lipodystrophy and harbored 6 different *POLD1* variants, including 3 novel variants that have not been reported in the literature before. Furthermore, we reviewed all the cases reported in the literature so far and compared the phenotypic differences among those with *POLD1* p.Ser605del and missense variants, including our cases.

## Material and methods

All patients and/or their parents gave written informed consent. The study protocol was approved by the institutional review boards of the University of Texas Southwestern Medical Center (UTSW) and the National Institutes of Health (NIH). The institutional review board of the Mayo Clinic provided exemption for anonymous reporting of the patient, FPL446.3. Based on the initial phenotypic presentation of the patients with various subtypes of lipodystrophies to us, we assigned them into various categories, such as familial partial lipodystrophy (FPL); MDPL; and miscellaneous type of lipodystrophy (MISC).

### Genetic sequencing

Whole-exome sequencing (WES) of patients MDP 100.3, 200.5, 400.3, 600.4, and 700.3 was performed at the University of Washington's Center for Mendelian Genomics. WES of patients FPL238.3, FPL459.3, FPL460.24, and MISC1100.1 was performed using the IDT xGen Exome capture kit on the Illumina platform with the sequencing read length 2 × 150 base pairs at UTSW. DNA samples from buccal swabs of patient MDP1400.3 and her parents underwent WES at GeneDx. DNA from a blood sample of patient FPL190.3 was sequenced by Fulgent Diagnostics for candidate gene, *POLD1,* using next-generation sequencing, followed by Sanger sequencing for the specific *POLD1* variant in his 2 affected offsprings, FPL190.15 and FPL190.16, at UTSW. FPL446.3 and her parents underwent whole-genome sequencing at Baylor Genetics.

### Statistical analysis

The comparisons of demographic, clinical features, and metabolic complications between carriers of *POLD1* p.Ser605del and missense variants were made by the Fisher exact test for categorical variables and the Wilcoxon rank sum test for continuous variable using R (v4.4.2). Multiple testing correction was performed using the Benjamini-Hochberg procedure.

### Case descriptions

Clinical features of 5 patients (MDP 100.3, 200.5, 400.3, 600.4, and 700.3) were previously reported (1), and only their key findings and follow-up information are presented. *POLD1* variants detected in our patients, their in silico functional prediction scores, as well as the allele frequencies in large databases are shown in Table 1. Salient clinical features, body composition, and metabolic variables of all the patients are summarized in Table 2 and Supplementary Table S1 (19), and only other features are mentioned in the descriptions that follow. The age of onset of hearing loss was provided by the patients or their parents as part of historical information and was also included in the lipodystrophy questionnaire. Most of the patients were diagnosed with hearing loss during childhood. We do not have audiological data at the diagnosis of hearing loss or during follow-up. However, for the majority of patients we have clear documentation that they are using hearing aids (MDP1400.3, FPL190.3, MDP100.3, MDP200.5, MDP300.4, MDP400.3, MDP600.4, MDP700.3), strongly suggesting objectively confirmed hearing loss.

**Table 1:** DNA Polymerase δ 1, catalytic subunit variants in our patients with mandibular hypoplasia, deafness, progeroid features, and lipodystrophy syndrome

Variant					In silico prediction*^c^*					Minor allele frequency		
Nucleotide change*^a^*	Amino acid change*^a^*	ID	Pathogenicity classification*^b^*	Patients	AlphaMissense	REVEL	EVE	CADD	GERP	GnomAD v4.1.0	UK Biobank	All of US
c.75delT	p.Asp25Glufs*16	rs772855121	VUS	FPL238.3	—	—	**—**	**—**	**—**	**6.26E−07**	**0**	**0**
c.1519C>T	p.Arg507Cys	rs878854523	VUS	FPL190.3, 190.15, 190.16, FPL459.3	0.992	0.75	**0.92**	**33**	**3.3**	**0**	**0**	**0**
c.1520G>A	p.Arg507His	rs2122336391	VUS	FPL446.3	0.986	0.49	**0.89**	**33**	**4.36**	**0**	**1.02E−06**	**0**
c.1812_1814delCTC	p.Ser605del	rs398122386	Pathogenic	MDP100.3, 200.5, 400.3, 600.4, 700.3, MISC1100.1	—	—	**—**	**—**	**—**	**6.26E−07**	**0**	**0**
c.2343G>C	p.Trp781Cys	rs2122450133	VUS	FPL460.24	0.7	0.41	**0.19**	**25.8**	**3.93**	**0**	**0**	**1.21E−06**
c.3185A>G	p.Gln1062Arg	rs2039339156	Pathogenic	MDP1400.3	0.961	0.33	**0.77**	**26**	**3.06**	**0**	**0**	**0**

Abbreviations: ACMG, American College of Medical Genetics and Genomics; CADD, Combined Annotation-Dependent Depletion; GERP, genomic evolutionary rate profiling; VUS, variant of uncertain significance.

^*a*^Mapped to NM_002691.4

^*b*^ACMG-based pathogenicity classification using Franklin by Qiagen (https://franklin.genoox.com/clinical-db/home).

^*c*^AlphaMissense (26), REVEL (25), and EVE (24) all assign a score between 0 and 1 to indicate the probability of a variant being pathogenic, with higher scores indicating higher likelihood of disease causing. The Phred-like CADD score (–10 × log_10_(%)) assigns a score range from 1 to 99, with higher scores indicating higher likelihood of disease causing. For example, a score greater than 20 indicates a variant is predicted to be in the top 1% of deleterious variants (27). The GERP score (23) range is −12.3 to 6.7 with higher scores indicating high constraint of sequences.

**Table 2:** Clinical phenotypes of mandibular hypoplasia, deafness, progeroid features, and lipodystrophy patients with a heterozygous DNA polymerase δ 1, catalytic subunit variant

	FPL238.3	FPL446.3	FPL190.15	FPL190.16	FPL190.3	FPL459.3	MDP100.3	MDP200.5	MDP400.3	MDP600.4	MDP700.3	MISC1100.1	FPL460.24	MDP1400.3
Amino acid change	p.Asp25Glufs*16	p.Arg507His	p.Arg507Cys	p.Arg507Cys	p.Arg507Cys	p.Arg507Cys	p.Ser605del	p.Ser605del	p.Ser605del	p.Ser605del	p.Ser605del	p.Ser605del	p.Trp781Cys	p.Gln1062Arg
cDNA variant	c.75delT	c.1520G>A	c.1519C>T	c.1519C>T	c.1519C>T	c.1519C>T	c.1812_1814del	c.1812_1814del	c.1812_1814del	c.1812_1814del	c.1812_1814del	c.1812_1814del	c.2343G>C	c.3185C>T
Age, y	55	27	45	34	65	33	63	51	12	21	18	16	34	16
Sex	F	F	M	F	M	F	M	M	F	M	M	F	F	F
Race	White	White	White	White	White	White	White	White	White	White	White	NA	White	White
Lipodystrophy	+ (PL)	+ (PL)	+ (PL)	+ (PL)	+ (PL)	+ (PL)	+ (GL)	+ (GL)	+ (PL)	+ (GL)	+ (GL)	+ (PL)	+ (PL)	+ (PL)
Telangiectasia	+	NA	NA	NA	NA	NA	NA	+	+	−	+	+	−	NA
Bird-like facies	NA	−	+	−	+	NA	NA	+	+	+	+	+	−	+
Mandibular hypoplasia	−	−	NA	−	+	NA	+	+	+	+	NA	NA	−	−
Dental overcrowding	NA	−	NA	NA	+	NA	+	+	+	+	+	NA	−	−
High-pitched voice	NA	NA	NA	NA	NA	NA	+	+	NA	NA	NA	NA	−	NA
Deafness (age of onset, y)	+ (childhood)	+ (8)	+ (?)	+ (?)	+ (21)	NA	+ (18)	+ (15)	+ (<10)	+ (15)	+ (0)	NA	+ (32)	+ (0)
Lack of breast development	−	−	NA	−	NA	−	NA	NA	+	NA	NA	−	−	+
Graying or loss of hair	NA	−	NA	−	NA	NA	NA	NA	NA	NA	NA	−	+	NA
Diabetes mellitus (age of onset, y)	+ (33)	+ (22)	+ (28)	+ (18)	+ (?)	−	+ (24)	−	−	−	−	−	−	−
Hypertriglyceridemia, (age of onset, y)	+ (36)	+ (22)	+ (28)	+ (18)	+ (?)	+ (?)	+ (26)	+ (7)	+ (12)	+ (21)	+ (15)	−	+ (34)	+ (15)
Hepatomegaly	NA	+	NA	NA	NA	NA	−	−	−	−	+	NA	+	+
Hepatic steatosis	NA	+	NA	NA	+	+	−	−	−	NA	+	NA	NA	+
Hypogonadism, male	NA	NA	−	NA	−	NA	+	+	NA	+	+	NA	NA	NA
Amenorrhea, female	−	−	NA	−	NA	−	NA	NA	NA	NA	NA	−	−	+
Joint contractures	−	−	NA	NA	NA	−	+	+	+	+	+	NA	−	+
Osteoporosis	NA	NA	NA	−	NA	NA	−	+	+	NA	+	NA	NA	+
Skin tightness	−	−	NA	NA	NA	+	NA	+	+	+	NA	+	−	−
Callus	NA	−	+	+	NA	NA	+	−	+	NA	NA	NA	−	−
Recurrent infections	NA	NA	+	+	+	NA	−	+	NA	NA	NA	NA	−	−
Leg cramps	NA	NA	+	+	+	NA	NA	NA	NA	+	NA	+	NA	−

Age at last follow-up is given.

Abbreviations: +, yes or present; −, no or absent; BMI, body mass index; cDNA, complementary DNA; F, female; GL, generalized lipodystrophy; M, male; NA, not applicable or not available; PL, partial lipodystrophy.

The previous paper by Shastry et al (1) provided detailed information on male hypogonadism. All 5 male patients (MDP100.3, 200.5, 500.4, 600.4, and 700.3) had hypogonadism requiring testosterone replacement and undescended testes (see Supplementary Table S1 (19)) and had the *POLD1* heterozygous p.Ser605del variant.

#### MDP100.3 (p.Ser605del)

This 63-year-old White man has been previously reported (1, 20, 21). His atrophic undescended testes were surgically removed at age 12 years (1). He had testicular hypoplasia and was started on testosterone replacement therapy at age 14 years.

#### MDP200.5 (p.Ser605del)

Since the previous reports (1, 22), this 51-year-old White man has had severe callous formation in the heels resulting in pressure ulcers. He had more than 10 episodes of osteomyelitis due to chronic wounds and had to undergo left leg amputation at age 51 years. He has hypogonadism and underwent a testicular biopsy at age 8 years showing immature testis with fibrosis, and rudimentary testes with growth arrest of seminiferous tubules and markedly reduced spermatogonia. His serum follicle-stimulating hormone (FSH) level was 2.7 IU/L (normal range, 2.0-10.0 IU/L), luteinizing hormone (LH) level was 3.4 IU/L (normal range, 1.2-8.6 IU/L), and testosterone was 0 ng/dL (normal range, 10-20 ng/dL for prepubertal males). He has received testosterone replacement therapy since age 8 years. He had an abnormal oral glucose tolerance test at age 46 years but was never diagnosed with diabetes mellitus. He was diagnosed with hypertriglyceridemia during his teens. He was diagnosed with hearing loss and has been wearing hearing aids since age 19 years.

#### MDP400.3 (p.Ser605del)

This 12-year-old White girl from Canada has been previously reported (1). No reproductive endocrine laboratory data were available for her.

#### MDP600.4 (p.Ser605del)

This 21-year-old Hispanic man has been previously reported (1). He had primary hypogonadism and undescended testes that were surgically removed, and was thereafter started on testosterone replacement therapy.

#### MDP700.3 (p.Ser605del)

This 18-year-old White young man from Germany has been previously reported (1). He underwent an orchidopexia during childhood and no testicles could be found. His baseline serum testosterone level was less than 0.2 µg/L (low) with a FSH level of 35.40 IU/L (normal range, 0.45-10.46 IU/L) and LH level of 3.83 IU/L (normal range, 0.48-7.93 IU/L). He has been on testosterone replacement since age 15 years.

#### MDP1400.3 (p.Gln1062Arg)

**Figure 1:**
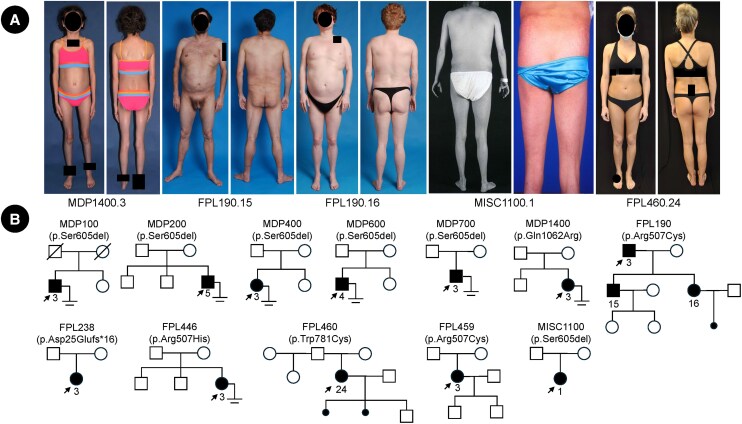
Clinical features and pedigrees of patients with DNA polymerase δ 1, catalytic subunit (*POLD1*) variants. A, Photographs of the affected patients. MDP1400.3 Anterior and posterior views of the 16-year-old White girl showing marked loss of subcutaneous fat from the upper and lower extremities and anterior trunk with increased muscularity. FPL190.15 Anterior and posterior views of the 45-year-old White man showing marked loss of subcutaneous fat and increased muscularity from the upper and lower extremities. Rectus abdominis is prominent due to lack of subcutaneous fat from the anterior abdomen. FPL190.16 Anterior and posterior views of the 34-year-old White woman showing marked loss of subcutaneous fat from the upper and lower extremities, particularly from the buttocks, with increased muscularity. MISC1100.1 Posterior views of the 16-year-old White girl showing loss of subcutaneous fat from the arms and legs. She has red colored telangiectasia on the lateral side of abdomen and trunk and on lower extremities. FPL460.24 Anterior and posterior views of the 34-year-old White woman showing moderate loss of subcutaneous fat and increased muscularity from the upper and lower extremities. B, Pedigrees of the patients with mandibular hypoplasia, deafness, progeroid features, and lipodystrophy (MDPL). Circles denote female patients, and squares denote male patients. Filled black symbols represent affected individuals with MDPL carrying the heterozygous *POLD1* variant, which is listed under the pedigree number. The white unfilled symbols indicate unaffected individuals. The number under the symbol indicates the pedigree number. Slanted arrows indicate the probands. Diagonal line across the symbol indicates deceased individual. Black dots indicate premature termination of pregnancy.

This 16-year-old White girl had a short stature and low weight since age 6 years. She was noted to have thin extremities with loss of subcutaneous fat, mandibular hypoplasia, and progressive sensorineural hearing loss since birth for which she was wearing hearing aids (Fig. 1). Puberty was delayed and she was diagnosed with primary ovarian insufficiency for which she used estradiol transdermal patches. Her serum estradiol level was less than 5 pg/mL (normal range, 12.5-211 pg/mL, depending on the phase of menstrual cycle), FSH levels were 58.8 and 75.9 IU/L (normal range, 0.9-9.9 IU/L for girls age 16-17 years), and LH levels were 16.4 and 12.5 μIU/L (normal range, <26.4 μIU/L for girls age 16-17 years). She had elevated cholesterol at age 15 years that was well controlled with atorvastatin and had a decreased bone mineral density. She appeared younger than stated age with a short stature and had some learning disabilities. She had hepatomegaly 4 cm below the right costal margin and on ultrasound had hepatic steatosis.

#### FPL190.3 (p.Arg507Cys)

This 65-year-old White man was diagnosed with diabetes mellitus complicated by peripheral neuropathy and had high triglycerides and advanced atherosclerosis. He had a prominent, pitched nose, small mandible with an overbite, and crowding of the teeth. He was diagnosed with partial lipodystrophy with thin extremities but increased truncal fat. He reported muscle cramping at night. He had hypogonadism but with no loss of fertility and had 2 children. He took testosterone replacement. He has had colon polyps.

#### FPL190.15 (p.Arg507Cys)

This 45-year-old male offspring of FPL190.3 had a small, pinched nose and prominent eyebrows with normal eyelashes. He had partial lipodystrophy since age 24 years with loss of subcutaneous fat on the extremities and increased truncal fat and acanthosis nigricans in the groins, axillae, and the neck (see Fig. 1). He reported callouses on his soles. He developed acute pancreatitis due to hypertriglyceridemia at age 30 years and had diabetes mellitus and tuberous xanthomas on the knees and elbows. He developed recurrent pneumonia since age 8 years and reported chronic muscle cramping at night in the lower extremities, as well as in his hands with prolonged activity. He had 2 healthy daughters, age 5 and 7 years. He has had left foot and right leg amputation due to infected ulcers on the toes. He reports progressive decline of his hearing over the years.

#### FPL190.16 (p.Arg507Cys)

This 34-year-old female offspring of FPL190.3 had partial lipodystrophy at age 18 years with thin extremities and loss of subcutaneous fat, increased truncal fat, and a wide rib cage (see Fig. 1). She was diagnosed with diabetes mellitus and had hypertriglyceridemia. Her menarche was at age 14 years but she was diagnosed with polycystic ovary syndrome. She had 1 pregnancy that resulted in a miscarriage at age 19 years. She reported recurrent upper respiratory infections and frequent cramping of the lower extremities at night. She was diagnosed with thyroid carcinoma and underwent total thyroidectomy at age 33 years. She reports difficulty hearing but has not had an audiological evaluation.

#### FPL238.3 (p.Asp25Glufs*16)

This 55-year-old White woman with partial lipodystrophy had diabetes mellitus and is on insulin therapy. She also has had hyperlipidemia and obesity since age 4 years with a disproportionate fat distribution with decreased subcutaneous fat on her arms and legs and increased fat around her chest, neck, abdomen, and face. She underwent hysterectomy for heavy menstrual bleeding with fibroids at age 54 years. She reported that her father had a similar fat distribution.

#### FPL459.3 (p.Arg507Cys)

This 34-year-old White woman has had partial lipodystrophy since a young age. She had thin extremities and facial lipoatrophy but started to develop subcutaneous and visceral fat deposition after pregnancy at age 24 years. She had delayed menarche with irregular menstrual cycles, but not infertility. She did not have hearing loss. Her mother was also diagnosed with partial lipodystrophy, and one of her daughters is noted to be thin with a lack of subcutaneous fat.

#### MISC1100.1 (p.Ser605del)

This 16-year-old White girl with lipodystrophy first noted minimal subcutaneous fat on upper and lower extremities up to buttocks at age 12 years, but the trunk and face appeared normal (see Fig. 1). She had telangiectasias over the extremities (biopsy consistent with telangiectasia macularis eruptiva perstans). She had small, flat breasts. There was no history of hearing loss. She reported stiffness of the body and leg cramping that occurred several times per month. She was diagnosed with hypothyroidism at age 14 years for which she was taking levothyroxine 75 mcg daily. She had menarche at age 11 years with regular periods. She had normal pubic and axillary hair. She had loss of fat from the extremities and palms and soles, and also had bilateral plantar callouses.

#### FPL460.24 (p.Trp781Cys)

This 34-year-old White woman with partial lipodystrophy (see Fig. 1) had a history of psychiatric illness, an eating disorder, and was diagnosed with acute pancreatitis secondary to hypertriglyceridemia at age 34 years. Her serum triglycerides remained elevated, despite treatment with fenofibrate, fish oil, and lifestyle changes. She did not have progeroid features or mandibular hypoplasia. She had difficulty hearing and ringing in the ears. She reported prominent veins on the arms and legs and acanthosis nigricans on the neck, axillae, and groin. She had premature graying and loss of hair. She had small breasts, and she underwent breast augmentation at age 21 years. She had menarche at age 12 years and had regular menstrual periods. She had 2 pregnancies; both were surgically terminated. She had increased facial and body hair.

#### FPL446.3 (p.Arg507His)

This 27-year-old White woman noted muscular appearance at age 10 to 12 years. She had severe menorrhagia from age 13 years and had intermittently been on oral contraceptives. She was diagnosed with diabetes mellitus and severe hypertriglyceridemia at age 22 years with blood glycated hemoglobin A_1c_ of 12.1% and serum triglycerides of 5770 mg/dL. She was started on metformin 1000 mg twice daily and gemfibrozil 600 mg twice daily, and subsequently insulin therapy (>200 units/day), and pioglitazone were added. Six months after the initiation of semaglutide, titrated to 1 mg weekly after 2 months, there was marked improvement in glucose control along with 12-kg weight loss, and she was able to discontinue insulin therapy. Hypertriglyceridemia also improved with cessation of oral estrogens and improvement in glucose control. She had mild hepatic steatosis diagnosed on abdominal computed tomography imaging along with mild elevation in serum alanine transaminase and alkaline phosphatase. She also had hypertension requiring treatment with lisinopril 20 mg daily and hydrochlorothiazide 25 mg daily. She had no facial dysmorphia or dental crowding. She had marked paucity of subcutaneous fat over the extremities and anterior trunk. Slight excess of fat was noted over the dorsocervical region. Mild acanthosis nigricans was noted over the neck. Liver was palpable 2 to 3 cm below the right costal margin. She reported difficulty hearing at age 32 years. She had hair loss all over the scalp and graying of the eyebrow and some scalp hair in her 30s.

## Results

WES, whole-genome sequencing, and targeted sequencing for *POLD1* in our patients revealed the following potential disease causal variants: c.75delT (p.Asp25Glufs*16); c.1519C>T (p.Arg507Cys); c.1520G>A (p.Arg507His); c.1812_1814CTCdel (p.Ser605del); c.2343G>C (p.Trp781Cys); and c.3185C>T (p.Gln1062Arg) (see Table 1). Of these, c.75delT (p.Asp25Glufs*16), c.1520G>A (p.Arg507His), and c.2343G>C (p.Trp781Cys) are novel *POLD1* variants in MDPL patients. The conservation score genomic evolutionary rate profiling (GERP) (23), multiple in silico functional prediction scores (24-27), as well as the allele frequencies in large databases were annotated. All missense variants occurred at highly conserved sites, as shown by multiple species alignment (Supplementary Fig. S2 (19) with GERP scores greater than 3, and predicted to be functional with Combined Annotation-Dependent Depletion [CADD] scores greater than 25.0, though there was no functional experiment performed in this study), except that p.Ser605del and p.Gln1062Arg were documented as pathogenic in ClinVar; other variants were classified as variant of uncertain significance (VUS) using Franklin by Qiagen (https://franklin.genoox.com/clinical-db/home). Except for one family (FPL190), all of them were sporadic, isolated cases. In addition, in patient FPL446.3, a paternally inherited variant in *DSPP*:c.1297G>T, p.G433*, rs1181249113, was also noted. Defects in the *DSPP* gene are associated with dentinogenesis imperfecta and in some patients with sensorineural hearing loss. She did report history of periodontal disease; however, her father did not have any hearing loss. We did not find any pathogenic variants in other lipodystrophy-associated genes or in mitochondrial genes.

Overall, our patients presented with either generalized (n = 4) or partial (n = 10) lipodystrophy, often manifesting at a young age. In addition, hypertriglyceridemia (defined as serum triglyceride levels >150 mg/dL) was diagnosed in all but 1 case (n = 12/13), and 2 patients (FPL460.24, FPL190.15) developed acute pancreatitis secondary to extreme hypertriglyceridemia. Diabetes mellitus was mostly (n = 4/5, 80%) diagnosed in patients with p.Arg507Cys/His variants, although 2 other patients were diagnosed with diabetes mellitus as well; 1 at age 24 years and 1 at age 33 years. All male patients (n = 4/4) with the *POLD1* p.Ser605del variant had undescended testes, compared with none (n = 0/2) of the males with the p.Arg507Cys variant. Two male patients belonging to the FPL190 pedigree had normal fertility and had 2 offsprings each, and the female belonging to the FPL190 pedigree had 1 pregnancy that resulted in a miscarriage.

As shown in Supplementary Table S2 (19), of the 33 previously reported cases plus the 9 new cases in this study, most patients harbored the recurrent *POLD1* p.Ser605del (n = 26), 15 had a *POLD1* missense variant, and 1 patient had a *POLD1* frameshift mutation, (p.Asp25Glufs*16). A comparison of the phenotypes of the patients with p.Ser605del and missense variants are shown in Tables 3 and 4. The main rationale to group all missense variants was to ascertain whether they cause a milder form of MDPL compared to the most prevalent p.S605del variant. There was no difference in the ratio of males and females, age, birth weight, or body mass index (BMI) in the two groups. Compared with individuals harboring the *POLD1* missense variants, those with the p.Ser605del variant had a higher prevalence of facial features, including mandibular hypoplasia (57% vs 100%, respectively; *P* = .015), small mouth (36% vs 100%, respectively; *P* = .015), and crowded teeth (44% vs 91%, respectively; *P* = .046), and hypogonadism in males (0% vs 92%, respectively; *P* = .046). Lipodystrophy was present in all cases, irrespective of the type of *POLD1* variant. Skin fold measurements are shown in Supplementary Fig. S1 (19). There were no differences in the prevalence of metabolic manifestations such as hepatomegaly, hepatic steatosis, hypertriglyceridemia, and diabetes mellitus in the two groups. Sensorineural hearing loss was a common feature of both groups (71% vs 88%, respectively; *P* = .59). The ages of onset of hearing loss (7.0 vs 11.5 years, respectively; *P* = .67), diabetes mellitus (28.0 vs 23.0 years, respectively; *P* = .42), or hypertriglyceridemia (25.0 years vs 16.0 years, respectively; *P* = .42) were also not statistically different between those with missense and p.Ser605del variants (see Table 3). When we compared patients with *POLD1* p.Ser605del to those with *POLD1* p.Arg507 variants (p.Arg507His and p.Arg507Cys; n = 7), similar variables remained statistically different, although those with *POLD1* p.Arg507 variants were older (median age 20.5 y vs 45.0 y, respectively; *P* = .012), and had a higher BMI (15.6 kg/m^2^ vs 25.9 kg/m, respectively; *P* = .005), as well as having a lower frequency of prominent eyes (88.9% vs 16.7%, *P* = .048) (Supplementary Tables S3 and S4) (19). A beaked nose and crowded teeth were no longer significantly less frequently seen in *POLD1* p.Arg507 as compared to those with p.Ser605del variants.

**Table 3:** Comparison of anthropometric parameters and continuous variables in individuals with heterozygous DNA polymerase δ 1, catalytic subunit p.Ser605del and missense variants

	*POLD1*, p.Ser605del		*POLD1*, missense		
	n	Median (IQR)	n	Median (IQR)	*P*adj*^a^*
Age, y	26	20.50 (13.50-30.75)	15	33.00 (17.00-39.50)	.422
Birth weight, kg	19	3.30 (3.00-3.75)	7	3.30 (2.50-3.40)	.422
Height, m	24	1.59 (1.45-1.64)	15	1.62 (1.50-1.71)	.760
Weight, kg	24	38.70 (30.98-47.30)	15	48.40 (29.50-69.95)	.422
BMI, kg/m^2^	25	15.60 (14.60-18.30)	15	18.20 (13.37-25.75)	.422
Sensorineural hearing loss, age onset, y	20	11.50 (7.00-15.00)	7	8.00 (5.00-22.50)	≥.999
Diabetes mellitus, age onset, y	9	23.00 (20.00-28.00)	5	28.00 (22.00-43.00)	.422
Hypertriglyceridemia, age onset, y	7	16.00 (13.50-18.50)	6	25.00 (19.00-32.50)	.442

Abbreviations: BMI, body mass index; IQR, interquartile range; *POLD1*, DNA polymerase δ 1, catalytic subunit.

^*a*^Adjusted *P* value is calculated by using the Wilcoxon rank sum test, and multiple testing correction was made by the Benjamini-Hochberg procedure.

**Table 4:** Comparison of clinical features in subjects with DNA polymerase δ 1, catalytic subunit null (p.Ser605del) and missense variants

	*POLD1*, p.Ser605del		*POLD1*, missense		
	N affected/total	% affected	N affected/total	% affected	*P*adj*^a^*
Sex, female	14/26	53.8%	11/15	73.3%	.568
Mandibular hypoplasia	24/24	100.0%	8/14	57.1%	.015
Graying or loss of hair	3/14	21.4%	2/9	22.2%	≥.999
Sensorineural hearing loss	21/24	87.5%	11/14	78.6%	.725
Prominent eyes	8/9	88.9%	4/11	36.4%	.081*^b^*
Beaked nose	25/25	100.0%	9/12	75.0%	.081*^b^*
High-pitched voice	11/17	64.7%	3/6	50.0%	.725
Small mouth	12/12	100.0%	4/11	36.4%	.015
Crowded teeth	21/23	91.3%	4/9	44.4%	.046
Lipodystrophy	26/26	100.0%	14/14	100.0%	≥.999
Joint contractures	15/16	93.8%	4/11	36.4%	.019
Telangiectasis	15/21	71.4%	2/5	40.0%	.568
Skin tightness	19/20	95.0%	5/9	55.6%	.081*^b^*
Hepatomegaly	4/14	28.6%	4/7	57.1%	.568
Hepatic steatosis	10/15	66.7%	8/10	80.0%	.725
Diabetes mellitus	9/26	34.6%	8/14	57.1%	.455
Hypertriglyceridemia	15/22	68.2%	12/15	80.0%	.691
Pancreatitis	0/6	0.0%	3/9	33.3%	.478
Osteopenia/osteoporosis	11/21	52.4%	4/6	66.7%	.725
Poor breast development, female	6/7	85.7%	4/9	44.4%	.371
Hypogonadism, male	11/12	91.7%	0/3	0.0%	.046
Amenorrhea, female	2/10	20.0%	3/8	37.5%	.725
Cryptorchidism, male	9/11	81.8%	2/4	50.0%	.699

Abbreviation: *POLD1*, DNA polymerase δ 1, catalytic subunit.

^*a*^Adjusted *P* value is calculated by using the Fisher exact test, and multiple testing correction was performed using the Benjamini-Hochberg procedure.

^*b*^Nominal *P* value of these variables was less than .03.

## Discussion

MDPL is an autosomal dominant disorder characterized by mandibular hypoplasia, deafness, progeroid features, and lipodystrophy. It is an extremely rare syndrome with 33 cases reported in the literature. In this case series, we report a cohort of 14 patients (9 of whom have not been reported previously) with different features of MDPL likely to be caused by 6 different heterozygous *POLD1* variants. Although the *POLD1* variants typically occur de novo, 1 of our families had a confirmed autosomal dominant inheritance pattern, and 2 additional patients had family histories suggesting autosomal dominant inheritance.

**Figure 2:**
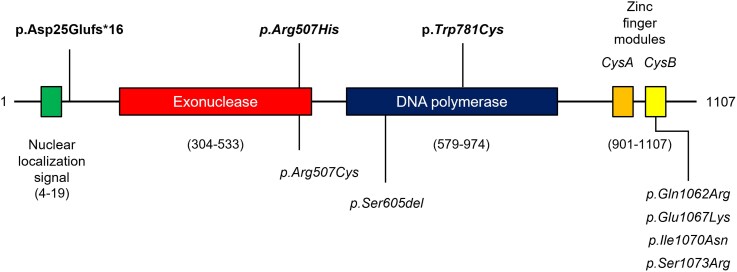
Schematic representation of the functional structure of DNA polymerase δ 1, catalytic subunit (POLD1) and variants associated with mandibular hypoplasia, deafness, and progeroid features associated with lipodystrophy (MDPL). POLD1 is composed of 1107 amino acids and includes several distinct domains: a putative nuclear localization signal (amino acids 4-19), an exonuclease domain (amino acids 305-533), a DNA polymerase active site (amino acids 579-974), and a zinc finger domain (amino acids 901-1107), which contains 2 conserved cysteine-rich metal-binding motifs (CysA and CysB). The position of the potential disease-causing variants identified in patients with MDPL are indicated, including the 3 novel variants reported in this study: p.Asp25Glufs*16, pArg507His, p.Trp781Cys in bold font.

*POLD1* encodes catalytic subunit (p125) of DNA polymerase δ (Pol δ), which is essential for lagging strand synthesis both with 5′- to 3′-polymerase activity and 3′- to 5′-exonuclease activity during DNA replication. It is also critical for proofreading during DNA replication through its exonuclease activity, maintaining genomic stability (28-30). POLD1 is composed of 1107 amino acids (31) and includes several distinct domains: a putative nuclear localization signal (amino acids 4-19), an exonuclease domain (amino acids 305-533), a polymerase active site (amino acid 579-974), and a zinc finger domain (amino acids 901-1107), which contains 2 conserved cysteine-rich metal-binding motifs (CysA and CysB) (7, 30, 32) (Fig. 2).

The most common pathogenic variant associated with MDPL is the heterozygous in-frame deletion c.1812_1814delCTC (p.Ser605del) (3, 7, 14-18). The p.Ser605del variant affects the polymerase active site of DNA polymerase δ, resulting in a loss of function of POLD1 (2). Five other missense variants that have previously been described, including p.Arg507Cys (3, 16) in the exonuclease domain; and p.Gln1062Arg (15, 17), p.Glu1067Lys (18), p.Ile1070Asn (7), and p.Ser1073Arg (14) in the CysB. Here, we report 3 additional novel variants resulting in an MDPL phenotype: one affecting the N-terminal domain that leads to a disruption in the protein's normal structure and function (p.Asp25Glufs*16), one in the exonuclease domain (p.Arg507His), and one affecting the polymerase active site (p.Trp781Cys). The 2 missense variants p.Arg507His and p.Trp781Cys occurred at conserved sites with GERP scores equal to 3.30 and 3.93, respectively, and were predicted to be functional with CADD scores equal to 33.0 and 25.8, respectively. Ensembl Variant Effect Predictor (VEP) (33) predicts the frameshift variant p.Asp25Glufs*16 as a “nonsense-mediated [messenger RNA] decay (NMD)-escaping_variant.” All 3 variants were classified as VUS using Franklin by Qiagen. However, there was no functional experiment performed in this study.

Although MDPL is a monogenic disorder, it has a heterogenous presentation with shared features as highlighted in the case presentations and Table 2, and not all patients express the full features of MDPL syndrome reported originally (1). Our comparison of clinical features in all patients described in the literature with the p.Ser605del and missense variants reveals that those with the p.Ser605del variant had typical MDPL as reported by Shastry et al (1). Patients with the missense variants, however, had reduced prevalence of mandibular hypoplasia and progeroid features and had a phenotype of FPL, rather than generalized lipodystrophy, which we therefore defined as “atypical” MDPL. They also had a lower prevalence of facial features, including prominent eyes, beaked nose, small mouth, and crowded teeth.

Sensorineural hearing loss was a common feature in both groups, with no difference in age of onset of hearing loss. The pretest probability of being MDPL, that is, finding a disease-associated variant in patient FPL446.3, was quite high given that she had sensorineural hearing loss at age 8 years even though she presented with FPL and severe metabolic derangements. Similarly, FPL460.24 also reported hearing loss at age 32 years. To the best of our knowledge, sensorineural hearing loss has not been reported in patients with FPLD2 due to *LMNA* variants and FPLD3 due to *PPARG* variants (34). In a thorough review of *LMNA* pathogenic variant–associated lipodystrophy by Besci et al (35), there was no mention of hearing loss. However, we have previously reported late-onset sensorineural hearing loss at age 31 years in a 53-year-old female proband with atypical progeroid syndrome and FPL with a heterozygous *LMNA* p.P4R variant, and in her 33-year-old affected son at age 23 years but not in her 22-year-old affected daughter (36).

The original description of the syndrome identified hypogonadism in affected males, with undescended testes and underdeveloped secondary sexual characteristics. Consistent with our prior observation (1), males with the *POLD1* p.Ser605del variant had a significantly higher frequency of hypogonadism, affecting all but one case as compared to males with missense variants. None of the reported females with the *POLD1* p.Ser605del variant had pregnancy. In contrast, one of our female patients with the p.Arg507Cys variant had one pregnancy that resulted in a miscarriage and another one with the same variant did have normal fertility with one healthy offspring. Another female with the missense p.Trp781Cys also had normal fertility: she had 1 daughter and 2 early-terminated pregnancies.

Manifestations of metabolic syndrome and endocrinopathies are also closely related to the syndrome's overarching phenotype, and are likely the result of lipodystrophy and premature cellular aging and genomic instability. Metabolic manifestations such as hepatomegaly, hepatic steatosis, hypertriglyceridemia, and diabetes mellitus were common, but equally distributed across the two groups, and there was no difference in age of onset of metabolic complications.

The mechanism through which the *POLD1* variants leads to the clinical variability in MDPL syndrome is largely unknown, but different variants affecting different domains of *POLD1* likely contribute to presentation. Weedon et al (2) showed that the p.Ser605del variant disrupts hydrogen bonds between the trinucleotide substrate and the catalytic aspartate residues, thus affecting the structure of the polymerase active site while leaving the exonuclease domain intact. They demonstrated that the p.Ser605del variant retained DNA binding capacity, but had no DNA polymerase activity. Moreover, the exonuclease activity of p.Ser605del was lower compared to wild type. Murdocca et al (10) demonstrated that fibroblasts from a patient with MDPL carrying the p.Ser605del variant exhibited a number of hallmark “aged” cellular phenotypes in vitro, and noted accelerated cellular senescence and a decline in cell growth and proliferation with a cell cycle arrest in G0/G1, a delayed recovery from DNA damage, indicating a compromised DNA repair mechanism, and accelerated telomere shortening. In contrast, Oh et al (37) reported 2 patients with isolated sensorineural hearing loss without syndromic features of MDPL, harboring *POLD1* variants p.Gly1100Arg and p.Ser197Hisfs*54. They demonstrated partial loss of polymerase activity with preserved proofreading, suggesting a possible relationship between the degree of enzymatic impairment and disease manifestations. No other functional studies have been conducted for the *POLD1* missense variants. Future studies with *POLD1* missense variants are needed to quantify the extent of functional impairment of polymerase and exonuclease activities.

While germline variants in the exonuclease domain affecting the DNA proofreading activity of *POLD1* are known to be a cause of hereditary cancer and adenomatous polyposis syndrome (38, 39), the exonuclease p.Arg507Cys variant was not considered pathogenic for a predisposition to cancer by the American College of Medical Genetics and Genomics/Association for Molecular Pathology (40), and our cohort did not seem to be affected by an increased incidence of malignancies, although no structured oncologic surveillance was performed.

Due to the rarity of the syndrome, one of the limitations of our study is the relatively small number of patients. A second limitation is that some of our patients were diagnosed at a younger age before full manifestations of some of the clinical features, but future follow-up will reveal the severity of the phenotype of MDPL in these patients.

In conclusion, this phenotypic comparison between lipodystrophy patients carrying *POLD1* variants shows a clear phenotypic variability between the p.Ser605del and missense variants, which more commonly result in atypical MDPL. Clinicians should pay specific attention to metabolic management and monitoring of patients diagnosed with MDPL.

## Data Availability

All datasets generated during and/or analyzed during the current study are not publicly available but are available from the corresponding author on reasonable request.

## Abbreviations

BMI: body mass index
CADD: Combined Annotation-Dependent Depletion
FPL: familial partial lipodystrophy
FSH: follicle-stimulating hormone
GERP: genomic evolutionary rate profiling
LH: luteinizing hormone
MDPL: mandibular hypoplasia, deafness, progeroid features, and lipodystrophy
MISC: miscellaneous type of lipodystrophy
*POLD1*: DNA polymerase δ 1, catalytic subunit
UTSW: University of Texas Southwestern Medical Center
VUS: variant of uncertain significance
WES: whole-exome sequencing
